# Supplementary material for: Evolution of research funding for neglected tropical diseases in Brazil, 2004–2020
Source: PLoS Negl Trop Dis. 2023 Mar 16;17(3):e0011134. doi: 10.1371/journal.pntd.0011134 (PMC10019684; doi:10.1371/journal.pntd.0011134)
Supplement: Attachment — Submitted filename: Response to reviewers.pdf [file pntd.0011134.s001.pdf]

Dear Editor,

We thank you for the opportunity to have our manuscript **PNTD-D-22-01150**, entitled "Evolution of research funding for neglected tropical diseases in Brazil, 2004-2020", evaluated for publication by PLOS Neglected Tropical Diseases. We are grateful to the reviewers for their time and effort and have implemented the suggestions to improve the manuscript, as described below.

Sincerely,

The Authors

Reviewer 1

Key Review Criteria Required for Acceptance

Methods

"It is my feeling that the methods selected are soundly robust to address the aimed objective".

Our response: *We would like to thank you for your consideration and time spent reviewing our manuscript.*

Results

"Results presented in tables and figures are quite detailed to allow the reader to follow the discussion."

Our response: *Thank you for your kind comment and assessment.*

Conclusions

"Yes, the conclusions are directly related to the results presented."

Our response: *Thank you for your kind comment.*

Editorial and Data Presentation Modifications?

"Gradient of blues in Figure 3 makes difficult to understand which organization is responsible for each percentage or disease. Try using different colors."

Our response: *Thank you for the suggestion. We have changed the color of the figure to make it easier to understand. Furthermore, the addition of another figure to the article means that Figure 3 is now Figure 4.*

Summary and General Comments

Comment 1: “The article addresses a very important issue, i.e., the shortage of funds for research in health in developing countries. This objective is higly relevant and interesting.”

Our response: *Thank you for your comment and the time taken to review our manuscript.*

Comment 2: “I believe authors used a robust methodology to proof their hypothesis and results are quite relevant.”

Our response: *Thank you for your comment. We believe that the results presented in our manuscript are relevant to different public and private stakeholders, including researchers, policy makers, health professionals and managers and funders, because they provide support for the monitoring and assessment of funding by the Brazilian Ministry of Health’s Department of Science and Technology as an example to other countries.*

Comment 3: “They clearly explain the somewhat confuse Brazilian governmental system of research funding. However, my concern is that the international reader may remain still confused - anyhow, one cannot blame authors for that.”

Our response: *Thank you for your comment. Indeed, the government funding system for research in Brazil is complex and can be difficult for international readers to understand. With this in mind, we have added Figure 1 to the Methods section to illustrate the public research funding process in Brazil and the governance system of the Ministry of Health’s Department of Science and Technology, the focus of our manuscript. We have also changed the wording of the context of the study (line 107 to 142) in the methods section to help readers more clearly understand the research funding system in Brazil, as follows:*

In Brazil, the National Science, Technology and Health Innovation System includes state and federal agencies and institutions that finance, monitor and evaluate research either individually or collectively (Fig 1A)[19]. In the present study, we analyzed funding for NTD research from the Ministry of Health’s Department of Science and Technology (MoH/DECIT).

In the federal sphere, within the Ministry of Health, DECIT is responsible for implementing National Policy on Science, Technology and Innovation in Health and coordinating health R&D via intersectoral partnerships for funding health research, particularly with: i) other Ministry of Health departments; ii) the Oswaldo Cruz Foundation (FIOCRUZ) – affiliated with the Ministry of Health and one of the main public institutions that conducts health research in Brazil and produces drugs and biopharmaceuticals for the SUS; iii) the Ministry of Science, Technology and Innovations (MCTI) – responsible for formulating and implementing National Policy on Science and Technology; iv) the Coordination for the Improvement of Higher Education

Personnel (CAPES) – affiliated with the Ministry of Education (MEC) and aimed at supporting professors and researchers via research grants and scholarships; v) national funding agencies (such as the National Council for Scientific and Technological Development – CNPq, a funding agency affiliated with the MCTI that funds scientific and technological research and innovation and helps train qualified human resources for research), as well as state (Research Support Foundations – FAPs, public institutions that finance scientific research in Brazilian states) and international funding agencies[15,16,20–22]. It is important to note that ST&I/H falls under the SUS, guaranteed by the 1988 Federal Constitution and Organic Health Law[23,24].

Research funding by DECIT can occur in three ways[25]: a) national open calls for research proposals: selection of research projects at the national level, with the collaboration of the CNPq or other funding agencies; b) state open calls/decentralized support: carried out through the research program managed by SUS (PPSUS), in which research projects at the state level are selected in partnership with the FAPs, CNPq, State Departments of Health and State Departments of Science and Technology, considering the particularities of each Brazilian state; c) Direct Hiring: aimed at addressing the strategic demands of the Ministry of Health or emergency situations in public health, whereby researchers are hired directly without going through a public selection process.

In order to establish research funding priorities in public calls for tender or direct hiring, DECIT uses the National Agenda of Health Research Priorities as a guide, which is based on SUS principles and the national and regional health needs of Brazilians[26]. Appraisal seminars are held to monitor and assess research supported by DECIT (Fig 1B).

Fig 1. Public research funding in Brazil. (A) Main funders of public research. (B) Governance System of health research funding in the Ministry of Health's Department of Science and Technology.

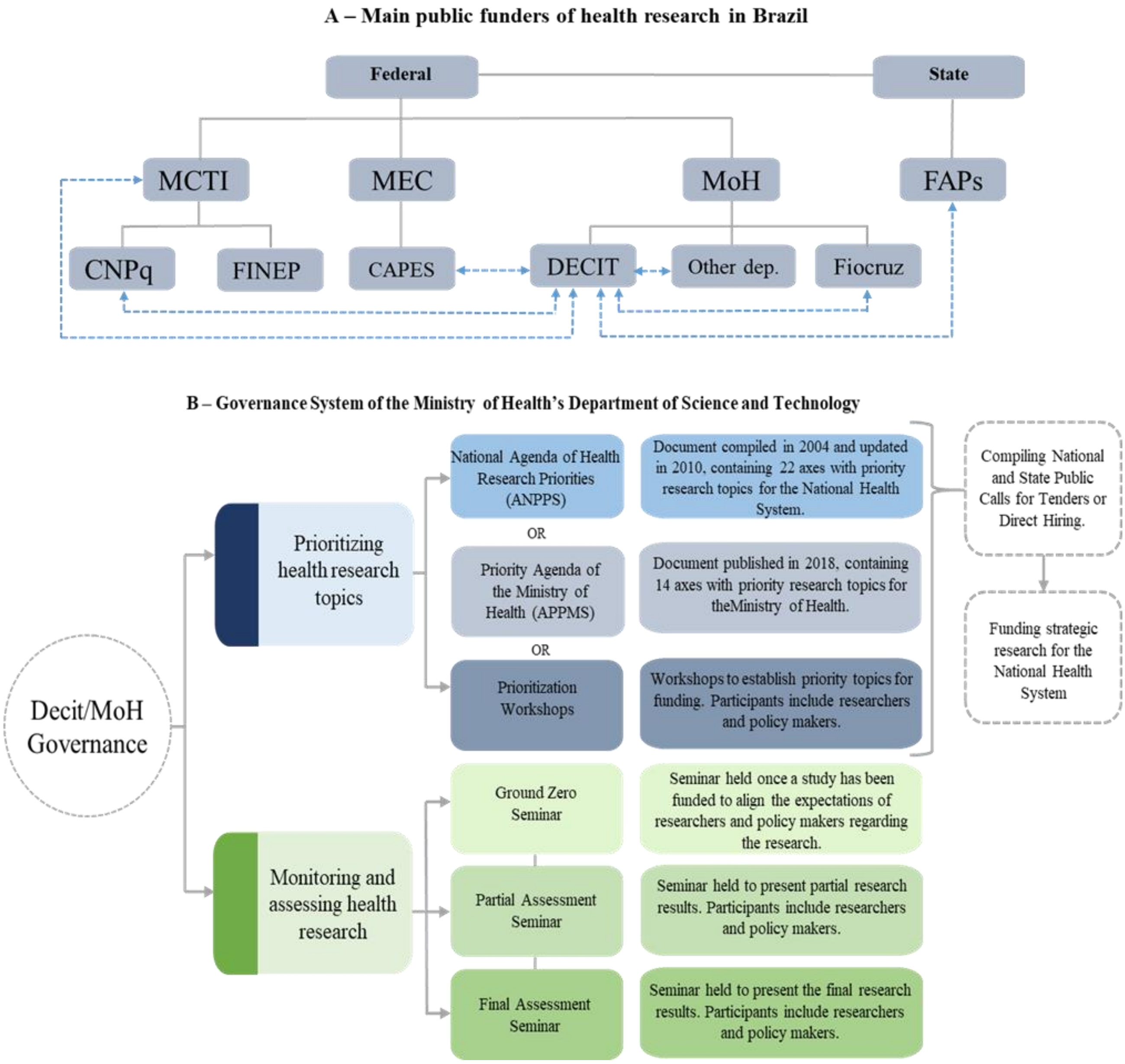

Legend: MCTI – Ministry of Science, Technology and Innovations; MEC – Ministry of Education; MoH – Ministry of Health; FAP – Research Support Foundations; CNPq – National Council for Scientific and Technological Development; FINEP - Funding Authority for Studies and Projects; CAPES – Coordination for the Improvement of Higher Education Personnel; DECIT – Department of Science and Technology; Other dep. – Other Ministry of Health departments; FIOCRUZ– Oswaldo Cruz Foundation.

Note: The blue arrows indicate partnerships with the Ministry of Health's Department of Science and Technology, the object of this study, to fund and conduct strategic health research for the SUS.

Source: Compiled by the authors based on the following publications: National Science, Technology and Innovation Strategy – 2016-2022 (available at: http://www.finep.gov.br/images/a-finep/Politica/16_03_2018_Estrategia_Nacional_de_Ciencia_Tecnologia_e_Inovacao_2016_2022.pdf); National Agenda of Health Research Priorities (available at: https://bvsms.saude.gov.br/bvs/publicacoes/agenda_nacional_prioridades_2ed_4imp.pdf); Research Priority Agenda of the Ministry of Health (available at: https://bvsms.saude.gov.br/bvs/publicacoes/agenda_prioridades_pesquisa_ms.pdf).

Comment 4: "Some of their results are most welcome, such as the imbalance of funding priority among top diseases and the information that biomedical research is by far the type of research mostly funded, in opposition to low percentage of clinical studies, which attests the difficulties of promoting clinical research out of the drug industry area."

Our response: *We agree with the reviewer's comment. The Brazilian Ministry of Health aims to strengthen clinical research structures and groups in Brazil. As a result, in collaboration with the Ministry of Science, Technology and Innovations (MCTI), it created the National Network of Clinical Research in Teaching Hospitals (RNPC) in 2005, in order to achieve an institutional clinical research model based on best research practices and the needs of the National Health System (SUS). The network was formalized in 2011 via Ministry of Health Ordinance 794 of April 13, 2011 (available at: https://bvsms.saude.gov.br/bvs/saudelegis/gm/2011/prt0794_13_04_2011.html) that aims to encourage the integration of clinical research centers in the country, promote the exchange of experiences between researchers and improve technological and scientific production.*

Presidential Decree 11287 was published on December 13, 2022, creating the Brazilian Clinical Research Network (RBPClin), to promote and encourage national scientific and technological development and innovation in clinical research (available at: https://in.gov.br/en/web/dou/-/decreto-n-11.287-de-13-de-dezembro-de-2022-450288909). RBPClin can be considered a progression of the RBPC by expanding the network's coverage in the country and the number of participating institutions, thereby representing an important advance in implementing national guidelines for clinical research in Brazil.

Additionally, in 2020, the Department of Science and Technology of the Ministry of Health published the Clinical Research Action Plan (available at: https://bvsms.saude.gov.br/bvs/publicacoes/plano_acao_pesquisa_clinica_brasil.pdf), aimed at improving Brazil's capacity to attract and develop clinical research to strengthen the National Health System.

Comment 5: "They also discuss this imbalance in terms of priority of funding and mortality/burden - that is, they complain that chikungunya, Chagas disease, malarial schistosomiasis and taeniasis/cysticercosis, which stand out in terms of prevalence and burden in Brazil, have received little research funding. However, an explanation for this may be the lack of submission of projects in these areas.

Our response: *We agree with your comment and have therefore included the following text in the Discussion section (line 420-430):*

It is believed that the small number of chikungunya studies funded is due to the date on which the disease was introduced in the country, since the first autochthonous cases were reported

in 2014[46]. For taeniasis/cysticercosis, the lack of consolidated public policies to tackle the disease may have resulted in its being "forgotten" in research in the field.

For other diseases, the dearth of research may be related to the introduction of other topics defined as priorities in public calls for tenders for study selection, prompting research groups to focus on topics with available funding. Additionally, the occurrence of malaria, for example, which is concentrated in the Brazilian Amazon Forest in Northern and Midwestern states[47], has made the disease a priority for these regions, meaning that often only researchers from these areas choose to study the topic.

Comment 6: "To some extent the article gives an idea that all governmental funding in health research is provided only by DECIT and its partners - that is not true. By large, most of the governmental health research funding is provided by other federal or state agencies (the partners alone!) that have higher budgets than DECIT. I dare to say that DECIT funds only just a very few number out of the overall health research conducted in the country. Perhaps authors should include a brief note to contextualize this fact."

Our response: *Thank you for your comment. Our object of analysis was funding for neglected tropical disease research, strategic to the Brazilian National Health System, provided by the Ministry of Health's Department of Science and Technology and its partners. However, other stakeholders also fund health research and as such, we have included Figure 1 in the Methods section, as well as a detailed description of the public research funding system in Brazil to make the text clearer and more easily understood (line 107 to 142).*

Comment 7: "I believe that the findings of this article are quite relevant to the research field in Brazil, no doubts. A possible constraint is that the issue is too much specific to our reality and may be difficult to foreign readers to understand the details of the organization of governmental funding for research in Brazil. However, in an overall view, the topic may be also of interest to the world community of health research."

Our response: *Thank you for your comment. As mentioned in comments 3 and 6, the research funding system in Brazil is complex and involves different stakeholders. We have therefore described the process in greater detail in the Methods section and included Figure 1, illustrating the main public funders of health research in Brazil and the governance system of health research in the Ministry of Health's Department of Science and Technology. We believe that despite the complexity of the model, it can be used as a reference and adapted to the reality of other countries, particularly low and middle-income countries.*

Reviewer 2

Key Review Criteria Required for Acceptance

Methods

"The use of the Prais-Winsten generalized line regression was applied in a time series considering the number of events (studies or $$) during the years. However, these events are strongly associated with the government changes. It is clear that each 3 – 4 years has one year with more funding (peak). The regression applied to the annual number is influenced by the fluctuation, as well the years almost without funding. My suggestion is to control the linear regression using splines for the government changes or just sum the total per period until the election of changes of management. At the end it would be possible to see the reduction in percentages."

Our response: *Thank you for your comment and suggestion. We agree with the reviewer and therefore included the variable "federal government management" in the temporal trend analysis as a spline. We found that although the funding trend remained stationary (annual variation rate of -5.7%; 95%CI -54.0 to 45.0), federal government management influenced investment in neglected tropical diseases research during the time period studied (p value < 0.05). The changes detailed here were made to the Methods section (line 205 to 229), as follows:*

Prais-Winsten generalized linear regression was used to analyze the temporal trend of funding, with a 5% significance level. Beta 1 coefficients ($\beta 1$) were estimated, with correction of the first-order temporal autocorrelation and respective confidence intervals of 95%, considering the year in which funding occurred as the dependent variable. The variable "federal government management" was also used, since management changes in the Brazilian federal government can influence research funding. In Brazil, federal government elections are held every four years and generally result in different policy and decision makers in federal organizations such as the Ministry of Health. These changes affect health research funding policies because political decisions by new Ministry of Health managers establish different priorities.

Thus, the different federal government managements were categorized as follows: i) Management 1 – 2004 to 2006; ii) Management 2 – 2007 to 2010; iii) Management 3 – 2011 to 2014; iv) Management 4 – 2015 to 2016; v) Management 5 – 2016 to 2018; vi) Management 6 – 2019 to 2020. Management 1 covered a three-year period that began in 2003 and therefore did not coincide with the start of data collection for our study (2004). Management 4 lasted two years because the president was impeached in 2016, when the vice-president took office for 2 years (2016 to 2018 – Management 5), and Management 6 covered two years, corresponding to the availability of data for this study (until 2020).

The estimated coefficients were used to calculate trends via the Annual Percent Change (APC) parameter and respective 95% confidence intervals (CI), calculated based on the following formula:

$$APC = [-1 + 10b1\ x\ 100; CI95\% = [-1 + 10b1min]\ x\ 100; 1 + 10b1\ \text{max}]\ x\ 100$$

A positive APC indicates an increasing trend for the time series analyzed, a negative value a decreasing trend, and no statistically significant difference is classified as stationary[29].

Results

"See the suggestions for the analysis."

Our response: *The suggestion has been accepted and temporal trend analysis redone.*

Conclusions

"Some parts of the conclusion do not depend on the results of this study. Should be based on the evolution analysis."

Our response: *Thank you for your comment. We have restructured the conclusion of the manuscript to comply with the points raised by the reviewer. The complete response to this question can be found in* ***Reviewer 2, comment 7.***

Editorial and Data Presentation Modifications?

It is important to correct the analysis, this way the results and discussion would be fitted.

Our response: *The temporal trend analysis was redone to comply with the points raised by the reviewer and the portions of the results and discussion sections that address the issue were restructured, as shown below:*

Results (line 249 to 254):

Analysis of the research funding trend during the study period showed a stationary trend, with an APC of -5.7% (95%CI -54.0 to 45.0). The amount invested in NTD research fluctuated during the period studied, declining sharply from 2006 to 2007, 2010 to 2011, 2014 to 2015 and 2016 to 2017, when changes occurred in the Brazilian federal government. These data indicate that changes in management influenced funding during the study period (p value < 0.05) (Fig 2B).

Discussion:

Line 355 to 356:

Although public funding for research on these diseases showed a stationary trend during this period, changes in the Brazilian federal government influenced the amount invested.

Line 385 to 392:

In addition to budget cuts, management changes in the Brazilian federal government affected investment in NTD research. It is important to underscore that although the same political party retained control of the federal government from 2004 to 2016, funding for NTD research fluctuated. This suggests that even with the same political party in power, changes occur that alter the direction of decision making on policies involving funding research with public resources. Governments play a key role in implementing policies to address NTDs on a national level, including establishing priorities that guide health research funding[2,36].

Summary and General Comments

Comment 1: "This is a very interesting study on the Evolution of research funding for neglected tropical diseases in Brazil, 2004-2021. The manuscript in general is original and relevant mainly for the stakeholders and managers of the research agency, government as well researchers. The sequence of the presentation is appropriate, but some points need to be reviewed."
Our response: *We would like to thank you for your consideration and time spent reviewing our manuscript.*

Comment 2: "Abstract: The results – Follow the topic results (reviewed)"
Our response: *The results portion of the Abstract was revised to reflect the modifications in temporal trend analysis, considering management changes in the Brazilian federal government, as follows (line 35 to 43):*

From 2004 to 2020, 1,158 studies were financed (purchasing power parity (PPP$) 230.9 million), with most funding aimed at biomedical research (81.6%) and topics involving dengue, leishmaniasis and tuberculosis (60.2%). Funding was stationary (annual percent change of -5.7%; 95%CI -54.0 to 45.0) and influenced by changes to the federal government. Research funding was lacking for chikungunya, Chagas disease, schistosomiasis, malaria and taeniasis/cysticercosis, diseases with a high prevalence, burden or mortality rates in Brazil.

Although the Ministry of Health had several budgetary partners, it was the main funder, with 69.8% of investments.

Comment 3: "Abstract: Conclusion – answer the objective – The recommendation is not the conclusion."

***Our response**: The conclusion of the Abstract has been restructured, as shown below (line 43 to 47):*

The study revealed that research funding for neglected tropical diseases has stagnated over the years and that diseases with a high prevalence, burden and mortality rate receive little funding. These findings demonstrate the need to strengthen the health research system by providing sustainable funding for research on neglected tropical diseases that is consistent with the population's health needs.

Comment 4: "Introduction: Line: 94 – 96 In Brazil, the Ministry of Health's Department of Science and Technology (DECIT) is currently the main funder of strategic research to improve the public health system and the health status of the population. However the reference refers to 2010 and before. Maybe better: …is usually the main…"

***Our response:** Thank you for your suggestion. We have included the updated reference on research funding by the Ministry of Health's* Department of Science and Technology*. In order to ensure better understanding of the paragraph and underscore its focus, which is research funding to improve the National Health System, we altered the text as follows (line 92 to 97):*

In Brazil, under the auspices of the Federal Government, the Ministry of Health's (MoH) Department of Science and Technology (DECIT in Portuguese) is one of the main funders of health research considered strategic in improving the National Health System (SUS)[14]. The goal of the department is to coordinate, articulate and induce health research, including on NTDs, within the scope of the National Science and Technology System[15,16].

Comment 5: "Results: The use of the Prais-Winsten generalized line regression was applied in a time series considering the number of events (studies or $$) during the years. However, these events are strongly associated with the government changes. It is clear that each 3 – 4 years has one year with more funding (peak). The regression applied to the annual number is influenced by the fluctuation, as well the years almost without funding. My suggestion is to control the linear regression using splines for the government changes or just sum the total per period until the election of changes of management. At the end it would be possible to see the reduction in percentages."

Our response: *Thank you for your comment. We agree with your suggestion and therefore redid the trend analysis considering the different management periods of the Brazilian federal government and found that although they influenced funding for neglected tropical diseases research in Brazil, the funding trend remained stationary. As such, we altered the wording of the results that refer to these data (line 249 to 254).*

Analysis of the research funding trend during the study period showed a stationary trend, with an APC of -5.7% (95%CI -54.0 to 45.0). The amount invested in NTD research fluctuated during the period studied, declining sharply from 2006 to 2007, 2010 to 2011, 2014 to 2015 and 2016 to 2017, when changes occurred in the Brazilian federal government. These data indicate that changes in management influenced funding during the study period (p value < 0.05) (Fig 2B).

Comment 6: "Discussion: The author could include some points related to prioritized NTDs as Dengue, TB and Leishmaniasis. For instance: international agenda as Bill & Melinda Gates support. The research group organized a network as TB in Brazil, inducing the MoH support. In the case of Dengue – the burden of disease; and for Leishmaniasis the delay in terms of treatment at the same time of the increased risk area of the incidence."

Our response: *Thank you for your suggestion. We have included issues related to dengue and leishmaniasis in the Discussion section, as shown below (line 398 to 408):*

In 2019, the global burden of dengue was 30.8 DALYs per 100,000 inhabitants, ranking 2nd in relation to other NTDs. In the same year, Brazil was ranked 6th in terms of the global dengue burden (18.74 DALYs per 100,000 inhabitants)[4].

Among other NTDs in Brazil, leishmaniasis was ranked 4th for disease burden in 2019[4] and its control remains a distant goal due to the involvement of multiple species, vectors and animal reservoirs, the social stigma surrounding the resulting skin lesions (deformities and scars), lack of accurate diagnostic methods and expansion into previously nonendemic areas[39–41]. Its treatment is also challenging because, despite several studies aimed at alternative therapies, existing options are limited by factors such as toxic side effects, causing poor treatment adherence, low efficacy and resistance to available drugs[42,43].

In regard to the suggestion to cite Rede TB as an example of tuberculosis research conducted with the support of the Ministry of Health, we have included the following text (line 464 to 475) in our Discussion. In it we highlight Rede TB as a success story of the partnership between the Department of Science and Technology and the Bill and Melinda Gates Foundation in research funding. In addition to Rede TB, we have also included the World Mosquito Program, another

research project funded by Department of Science and Technology and Bill and Melinda Gates Foundation that has achieved promising results.

Another success story is the international partnership with the Bill and Melinda Gates Foundation to finance NTD research, such as in the Brazilian Tuberculosis research Network (RedeTB) and World Mosquito Program, whose results have advanced the SUS.

RedeTB aims to provide scientific and technological training for the development of technologies and products to control tuberculosis. One of its achievements is the incorporation of the Xpert MTB/Rif test into the SUS, used to diagnose more than 60% of tuberculosis cases in Brazil[57,58].

The World Mosquito Program, present in 12 countries, is an initiative aimed at combatting arboviruses that replaces Aedes aegypti mosquitoes with Wolbachia mosquitoes, a bacteria capable of reducing the transmission of dengue, zika, chikungunya and yellow fever. Research in Brazil has achieved positive results, with a reduction in arbovirus infection rates in areas where Wolbachia mosquitoes have been released[59,60].

Comment 7: "Conclusion: Some parts of the conclusion do not depend on the results of this study. Should be based on the evolution analysis."

Our response: *Thank you for your comment. We have restructured the conclusion, as follows (line 497 to 520):*

It was concluded that while funding for NTD research remained stable, changes in the federal government influenced the amount invested during the time period studied. The lack of increased funding for NTD research may imply a decline in scientific production and the search for new knowledge that offers solutions to control these diseases.

Dengue, leishmaniasis and tuberculosis were the most studied diseases and received more funding, indicating that they are priorities for the federal government and researchers. On the other hand, few resources were available for studies on chikungunya, Chagas disease, schistosomiasis, malaria and taeniasis/cysticercosis, diseases with a high prevalence, burden or mortality rate in Brazil, which suggests that research funding does not reflect the epidemiological importance of these diseases in the country.

The most funding was allocated to biomedical research. Continued funding for biomedical research is important in obtaining answers to persistent problems involving NTDs; however, this finding demonstrates the need to expand the scope of funding to include other types of research, such as epidemiological studies, in healthcare services and clinics in order to assess the effectiveness of the surveillance strategies, treatments and diagnostics adopted and seek new sustainable solutions.

The Ministry of Health played a leading role in funding NTD research, but also collaborated with several budgetary partners, particularly FAPs. These intersectoral partnerships can contribute to maximizing the capture and use of financial resources.

Finally, the results of this study contribute to subsidizing assessments of governance structure in health research, which must be coherent and based on health needs in order to increase the capacity of health systems, services and policies and improve funding in a scenario of finite and limited resources.
